# Application of Artificial Intelligence for Surface Roughness Prediction of Additively Manufactured Components

**DOI:** 10.3390/ma16186266

**Published:** 2023-09-18

**Authors:** Temesgen Batu, Hirpa G. Lemu, Hailu Shimels

**Affiliations:** 1 Department of Aerospace Engineering, Ethiopian Space Science and Geospatial Institute, Addis Ababa P.O. Box 33679, Ethiopia; temesgen.batu@kiot.edu.et; 2 Center of Armament and High Energy Materials, Institute of Research and Development, Ethiopian Defence University, Bishoftu P.O. Box 1041, Ethiopia; 3 Department of Mechanical and Structural Engineering and Materials Science, University of Stavanger (UiS), 4036 Stavanger, Norway; 4 Department of Mechanical Engineering, College of Engineering, Addis Ababa Science and Technology University, Addis Ababa P.O. Box 16417, Ethiopia; hailu.shimels@aastu.edu.et

**Keywords:** surface roughness, roughness prediction, additive manufacturing, machine learning

## Abstract

Additive manufacturing has gained significant popularity from a manufacturing perspective due to its potential for improving production efficiency. However, ensuring consistent product quality within predetermined equipment, cost, and time constraints remains a persistent challenge. Surface roughness, a crucial quality parameter, presents difficulties in meeting the required standards, posing significant challenges in industries such as automotive, aerospace, medical devices, energy, optics, and electronics manufacturing, where surface quality directly impacts performance and functionality. As a result, researchers have given great attention to improving the quality of manufactured parts, particularly by predicting surface roughness using different parameters related to the manufactured parts. Artificial intelligence (AI) is one of the methods used by researchers to predict the surface quality of additively fabricated parts. Numerous research studies have developed models utilizing AI methods, including recent deep learning and machine learning approaches, which are effective in cost reduction and saving time, and are emerging as a promising technique. This paper presents the recent advancements in machine learning and AI deep learning techniques employed by researchers. Additionally, the paper discusses the limitations, challenges, and future directions for applying AI in surface roughness prediction for additively manufactured components. Through this review paper, it becomes evident that integrating AI methodologies holds great potential to improve the productivity and competitiveness of the additive manufacturing process. This integration minimizes the need for re-processing machined components and ensures compliance with technical specifications. By leveraging AI, the industry can enhance efficiency and overcome the challenges associated with achieving consistent product quality in additive manufacturing.

## 1. Introduction

Over recent decades, the manufacturing sector has gone through significant changes, yet it continues to play a critical role in the growth and development of both developed and developing countries [1]. It is considered the backbone of economies due to its contribution to economic growth, job creation, and technological advancement [2]. Additive manufacturing (AM) has gained acceptance among academics and the manufacturing sector as a powerful manufacturing tool. Recent research shows that it is more efficient than conventional production methods [3]. The manufacturing industry uses the term Additive Manufacturing to describe the process of fabricating physical objects from design data in a digital form by building them layer-by-layer. AM is a disruptive technology as it is not tied to individual production steps and does not require specific tools for each component, making it a universal production technology [4,5]. Numerous AM processes are currently available for commercial use. Various researchers have approached the classification of AM methods differently [6,7,8]. However, a widely accepted classification stems from the ASTM-F42 committee guidelines, which categorize AM into seven distinct groups [9]. These categories consist of vat photopolymerization (VP), material jetting (MJ), binder jetting (BJ), material extrusion (ME), sheet lamination (SL), powder bed fusion (PBF), and directed energy deposition (DED). The concise overview of all seven categories was presented in the paper [10].

The main advantage of additive manufacturing is that it can fabricate complex shapes and reduces material waste and production time (for some) [11,12,13,14]. Despite its advantages, additively manufactured products generally have poorer quality compared to those made with conventional manufacturing systems [15,16], primarily due to limitations in surface integrity [17]. Research indicates that in all additive manufacturing techniques, the improvement of surface roughness is a key objective. For example, vat photopolymerization (VP) and material jetting (MJ) yield parts with moderate surface roughness [10], whereas binder jetting (BJ), material extrusion (ME), sheet lamination (SL), powder bed fusion (PBF), and DED tend to result in parts with relatively poorer surface finishes [10]. Surface roughness is one of the factors encompassed by the concept of surface integrity. In Table 1, the surface roughness of most additively manufactured techniques is presented. 

The surface quality of additively manufactured components is influenced by a multitude of factors that interact in intricate ways. These factors include the choice of AM technique, powder characteristics, layer thickness, scanning strategy, and energy parameters specific to each technique. For instance, in Vat Photopolymerization (VP), parameters such as layer thickness, exposure time, and resin properties contribute to surface quality [40,41]. Similarly, in Material Jetting (MJ), drop size, print speed, and layer thickness impact the surface finish [40,42,43]. In Binder Jetting (BJ), binder saturation and layer thickness play a crucial role in achieving desired surface characteristics [44,45,46,47]. For material extrusion-based methods in fuse deposition modelling or fused filament fabrication, parameters such as nozzle diameter, layer height, and extrusion speed influence the surface roughness [48,49,50,51,52]. The terms fused deposition modeling (FDM) and fused filament fabrication (FFF) are two terms that are often used interchangeably to describe the same 3D printing process. However, FDM is a trademarked term by Stratasys, while FFF is a generic term used by the open-source community [53].

Powder Bed Fusion (PBF) [54,55] and Directed Energy Deposition (DED) [56] are also characterized by their unique process parameters that have direct implications on surface quality. While the powder bed fusion techniques such as SLS, SLM, DMLS, and L-PBF (Table 1) are fundamentally identical processes that use a laser to selectively melt or sinter a bed of powder material to create a 3D object, there exist some differences in the way these processes work, such as the type of laser used, the power of the laser, and the scanning strategy used to melt or sinter the powder. These differences can affect the quality of the printed part, including its surface roughness. In general, thinner layers result in smoother surfaces, but can also increase printing time and cost. The optimal layer thickness for a given process depends on several factors, including the size of the powder particles, the power of the laser, and the scanning speed used to melt or sinter the powder. Table 2 presents the process parameters of selected additive manufacturing techniques.

Furthermore, post-build treatments significantly contribute to the final surface quality of additively manufactured components [65,66]. For example, it can significantly improve the mechanical properties, i.e., surface roughness of the parts, making them more suitable for a wide range of applications [67]. Different post-processing techniques have been used by many researchers, such as shot peening (SP), laser shock peening (LSP), hot isostatic pressing (HIP), friction stir processing (FSP), and heat treatments (HT), to realize the better performance of the AM parts [66]. Various treatments, such as heat treatment, abrasive finishing, and chemical processes, can be applied to refine the surface texture and eliminate defects [68]. For instance, heat treatment can lead to grain growth and stress relief, affecting the overall surface roughness [63]. Abrasive finishing techniques, including sanding and polishing, can mitigate layer lines and irregularities, resulting in improved surface smoothness [68]. Chemical processes such as etching or electrochemical polishing can selectively remove material, enhancing surface finish [63]. Understanding the dependencies of surface roughness on these post-build treatments is crucial for achieving consistent and desirable surface qualities in additively manufactured components [69].

Surface roughness has been identified as one of the most significant factors affecting product quality, as stated in previous research [11]. For instance, Chan et al. [70] conducted a study to investigate the impact of surface roughness on product life and concluded that surface roughness leads to a reduction in product life expectancy. Moreover, surface roughness plays a role in the tribological behavior of surfaces [71], with rough surfaces experiencing faster wear compared to smooth surfaces. Therefore, it becomes crucial to predict and control the surface roughness of additively manufactured parts [64,72]. Additionally, surface roughness serves as an indicator for directly monitoring the mechanical characteristics of a workpiece, such as fatigue and surface friction, dimensional accuracy [73], and fracture resistance [74]. Understanding and managing surface roughness in manufacturing processes is essential for ensuring optimal product performance and longevity.

The impact of surface roughness on system performance is significant across multiple industries, such as aerospace [75,76], automotive [77,78], marine [79], semiconductor [80], chemical, wind energy [81], solar energy [82], food processing [83,84], and medicine [85,86]. Therefore, it is crucial to control the surface roughness of manufactured components. Thus, researchers give attention to predicting the surface roughness of additively manufactured components before printing/manufacturing the components. Predicting surface roughness in the manufacturing industry can lead to better quality control, cost savings, improved performance, and process improvement.

Different methods were used to predict the surface roughness of additively manufactured components, i.e., Taguchi-based regression models [87,88], statistical regression models [89], computational modeling (e.g., the FEM and Discrete Element Method (DEM)), [90] and machine learning methods [91]. There are several limitations of conventional (statistical) methods for predicting the surface roughness of additively manufactured components. Conventional methods for predicting the surface roughness of manufactured components often suffer from various limitations, such as a lack of flexibility, time-consuming nature, high cost, limited accuracy, limited applicability, inability to account for process parameter interactions, and limited understanding of underlying mechanisms [17,92].

However, the emergence of Artificial Intelligence (AI) approaches, including both machine learning and deep learning, has opened avenues to surmount these limitations. These AI approaches offer the potential to yield more precise and adaptable predictions of surface roughness. By harnessing these advancements in AI, particularly in machine learning, we can transcend the confines of traditional methods and attain the capability to design and fabricate high-quality components with unparalleled precision and efficiency. The flexibility, accuracy, and capacity to discern intricate patterns inherent in machine learning techniques empower us to overcome the challenges posed by conventional methods.

Machine learning methods have demonstrated remarkable capabilities in predicting complex patterns and relationships within diverse datasets [93,94]. Their strength lies in their ability to capture non-linear interactions among various process parameters—a challenge that conventional methods often struggle to address. By leveraging extensive datasets and sophisticated algorithms, machine learning models can unveil subtle correlations that might otherwise remain hidden. This not only enhances prediction accuracy, but also fosters a comprehensive understanding of the underlying factors shaping surface roughness. Moreover, the adaptability of machine learning techniques contributes to their predictive prowess [95]. Unlike rigid regression models, machine learning algorithms can continuously learn from new data, integrating fresh insights to refine their predictions. This adaptive nature proves especially advantageous in the realm of additive manufacturing, where process conditions and material properties can vary substantially. Consequently, machine learning empowers the development of predictive models that evolve alongside the manufacturing process, ensuring consistently precise predictions of surface roughness.

Deep learning, a subset of machine learning, adds a layer of sophistication to predictive modeling [96,97]. Neural networks within deep learning architectures automatically extract hierarchical features from raw data, enabling them to capture intricate patterns with remarkable precision. This capability proves invaluable when dealing with the complex structures and intricate relationships intrinsic to additive manufacturing processes. Deep learning models excel in analyzing intricate spatial and temporal interactions, offering insights into how diverse process parameters impact surface roughness across various production stages. The depth and complexity of deep learning models enable them to unearth nuanced relationships that may evade traditional methodologies. The hierarchical representation of features empowers deep learning models to untangle the underlying mechanisms driving surface roughness, contributing to a deeper comprehension of the manufacturing process itself. Consequently, deep learning not only delivers enhanced predictive capabilities, but also advances fundamental knowledge within additive manufacturing. The confluence of machine learning and deep learning with additive manufacturing holds tremendous potential to reshape quality assurance and process optimization.

Recently researchers gave great attention to AI for surface roughness prediction. Thus, this paper presents machine learning models or techniques used to predict the surface roughness of 3D printed parts. The remainder of the paper is organized as follows: Section 2 provides a method used to collect and select published data for the review. Section 3 introduces an overview of AI-based surface roughness predictions in AM. Section 4 presents a machine learning algorithm used for surface roughness predictions of AM parts. Section 5 presents a discussion on the trends, challenges, and future direction of applying AI algorithms for the surface roughness prediction of AM components. Section 6 provides conclusions and future work.

## 2. Methodology

To conduct this review, a rigorous and comprehensive methodology was employed. This section outlines the key steps undertaken to ensure a systematic and transparent approach to the review process. The systematic review followed the Preferred Reporting Items for Systematic Reviews and Meta-Analyses (PRISMA) guidelines [98] to ensure transparent and comprehensive reporting of the review process. The PRISMA flowchart was used to illustrate the study selection process and the number of studies included and excluded at each stage.

### 2.1. Search Strategy

The search strategy was developed to identify relevant studies on the application of artificial intelligence for surface roughness prediction of additively manufactured components. The following databases were searched: PubMed, IEEE Xplore, ScienceDirect, MDPI, and Google Scholar. The search terms used included variations and combinations of the following keywords: “artificial intelligence”, “machine learning”, “deep learning”, “surface roughness”, “prediction”, “additive manufacturing”, and “3D printing”. To consider all published papers related to the surface roughness prediction of additively manufactured components, the database search strategy focused on articles, proceedings, and books published in the journals listed above for the last 10 years.

### 2.2. Inclusion and Exclusion Criteria

The inclusion criteria used to select studies were defined as follows:

- Published materials in peer-reviewed journals or conference proceedings.
- Studies targeting the application of artificial intelligence techniques for surface roughness prediction in additively manufactured components.
- Studies that provided detailed methodology and results related to surface roughness prediction using artificial intelligence.
- Studies published in the English language.

The exclusion criteria for selecting studies were defined as follows:

- Studies that did not specifically address surface roughness prediction in additively manufactured components.
- Studies that focused solely on conventional manufacturing processes.
- Studies that were not available in full-text or were duplicates.
- Studies that were published in another language than English.

### 2.3. Inclusion and Exclusion Criteria

Two phases were used in the selection process: title/abstract screening and full-text assessment. The titles and abstracts of the identified studies are based on the inclusion and exclusion criteria. Any mismatch was resolved through discussion and consensus. In the full-text assessment phase, the reviewers thoroughly examined the selected articles to find out their eligibility for inclusion in the review.

### 2.4. Data Extraction and Analysis

Data extraction was done by the reviewer using a predefined data extraction form. The following information was extracted from each selected study:

- Objective of the study.
- Methodology employed (artificial intelligence techniques, algorithms, datasets used).
- Key findings and conclusions related to surface roughness prediction.
- Limitations and future directions identified by the authors.
- The extracted data were then synthesized and analyzed to identify common themes, trends, and patterns across the reviewed studies.

## 3. AI-Based Surface Roughness Prediction Overview

In this section, an overview of artificial intelligence and its application in predicting surface roughness is presented.

### 3.1. Artificial Intelligence Overview

Artificial intelligence is defined as a subset of IT applications that can capture information from its environment, comprehend, learn, interpret, and derive actions based on their implemented objectives [99]. In its strictest definition, AI stands for the imitation by computers of the intelligence inherent in humans [100]. AI can be classified into three categories based on its capabilities: General AI, Narrow AI, and Super AI. General AI represents a level of intelligence that can undertake any intellectual task with efficiency comparable to that of a human. This form of AI possesses a broader range of capabilities to adapt to various tasks [101]. Narrow AI, on the other hand, refers to AI systems that are designed to perform specific tasks with intelligence. It is the most common and currently available form of AI in the field. Super AI, also known as Artificial General Intelligence (AGI), signifies a level of intelligence in AI systems that surpasses human intelligence. It encompasses the ability to perform tasks more proficiently than humans, along with cognitive properties. Super AI is considered the outcome or extension of general AI [102].

**Figure 1:**
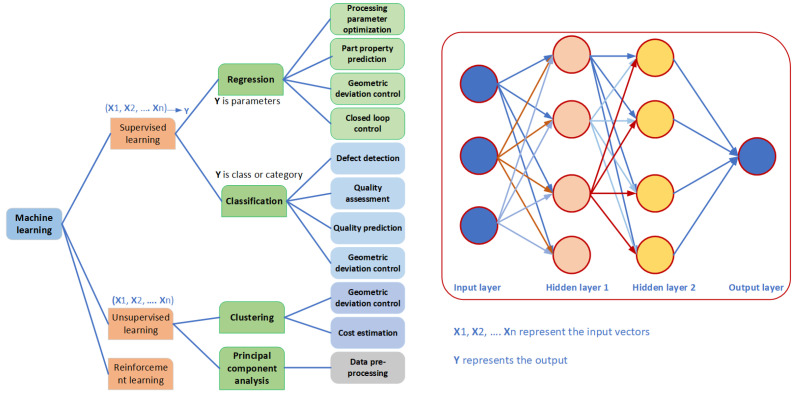
Taxonomy of machine learning applications in the AM domain. (Adapted from [104] an open-access article distributed under the terms of the Creative Commons CC-BY license).

Machine Learning, a prominent subset of AI, falls under the narrow AI category. It is characterized by its task-specific nature and focuses on a limited range of functionalities. Machine Learning algorithms are designed to learn from input data and enhance their performance in specific domains such as image recognition or natural language processing [103]. Machine Learning (ML) is a subfield of AI that emphasizes the development of algorithms and models enabling machines to learn from data and make predictions and decisions without explicit programming. In other words, ML involves training algorithms on extensive datasets to identify patterns and relationships, which are then utilized for making predictions or decisions [104]. ML’s primary objective is to analyze and learn from given datasets to perform tasks. It is classified into three categories: (1) supervised, (2) unsupervised, and (3) reinforcement learning. Figure 1 provides an overview of common ML approaches. In supervised learning, algorithms learn from labeled training data to predict outcomes, while unsupervised learning discovers relationships among features of interest using unlabeled data. Reinforcement learning enables the model to interact with the environment, learn, and take optimal actions that yield the highest rewards. ML finds application in regression, classification, and other tasks involving high-dimensional data, where training is based on learning from previous computations, and datasets can take various forms including audio signals [105], text [106], or images [107].

Machine learning plays a significant role in additive manufacturing, where it finds numerous applications. The integration of AI in additive manufacturing has the potential to revolutionize the field [108]. In a review by Meng et al. [109], various applications of machine learning in additive manufacturing were explored, such as process parameter optimization, part property prediction, geometric deviation closed-loop control, defect detection, quality assessment, etc. The review identified several primary areas of application machine learning for additive manufacturing (as illustrated in Figure 1).

### 3.2. Surface Roughness Prediction

#### 3.2.1. Definition of Surface Roughness and Its Measurements Techniques

**Figure 2:**
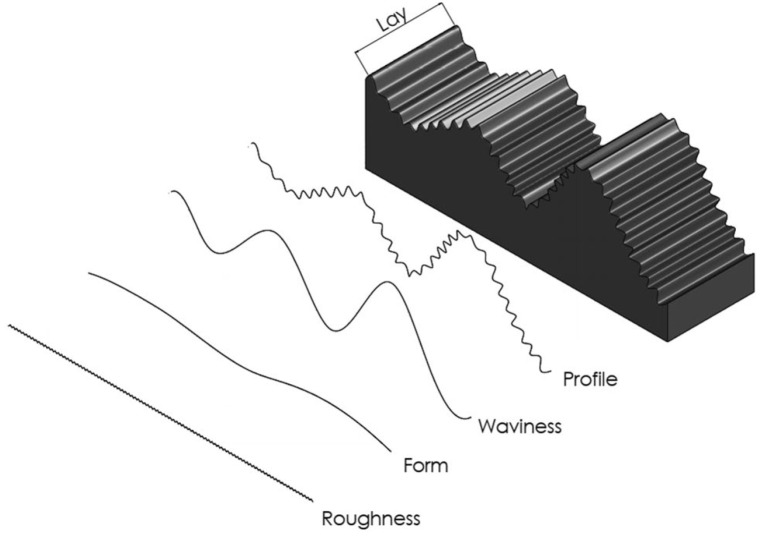
General spatial frequency components of additively manufactured surfaces [112] (an open-access article, © Int. J. Adv. Manuf. Technol., Springer) distributed under the terms of the Creative Commons CC-BY license).

Additively manufactured surfaces, as depicted in Figure 2, consist of profile, form, waviness, and roughness components, each with distinct origins and effects on product appearance and functionality. Waviness exposes machine vibrations, form arises from manufacturing system limitations, profile relates to layer-by-layer manufacturing, and roughness stems from printing and material removal errors. Waviness, akin to signal noise, emerges due to motion system planarity and deformations caused by weight or residual stress [110]. Process-specific factors, such as defects, thermal distortion, adhesion issues, support structure inadequacies, and post-processing deformation, also contribute to waviness [111,112].

Surface roughness, a crucial texture element, evaluates manufactured item quality by assessing topographical feature distribution. Diverse metrics are employed across industries, addressing uncertainty in 3D-printed product surface quality through multiple metrics for efficiency [113]. For instance, for the area surface roughness evaluation, the average area roughness (*S_a_*) and area root mean squared height (*S_q_*) are insensitive to measurement parameters [114]. Area height distribution skewness (*S_sk_*) effectively characterizes surfaces in SLM parts. Surface roughness impacts microstructures with peaks and valleys of varying heights, which is crucial as components miniaturize [115]. As such, research focuses on defining and controlling surface roughness during manufacturing.

Surface topography evaluations based on 3D-scanned image data of sample surfaces are categorized as linear measurements denoted as “R” or aerial surface measurements denoted as “S”, as per ISO 21920-2 [116] and ISO 25178-2:2021 [117] definitions. The roughness of a surface is often characterized by the area ratio, representing the real surface area relative to the ideal area of a smooth surface. Common height-based metrics, derived from Equations (1)–(10) in Table 3, describe surface roughness using linear profiles. Alternatively, area roughness parameters detailed in Table 4 capture roughness variations across surfaces.

**Figure 3:**
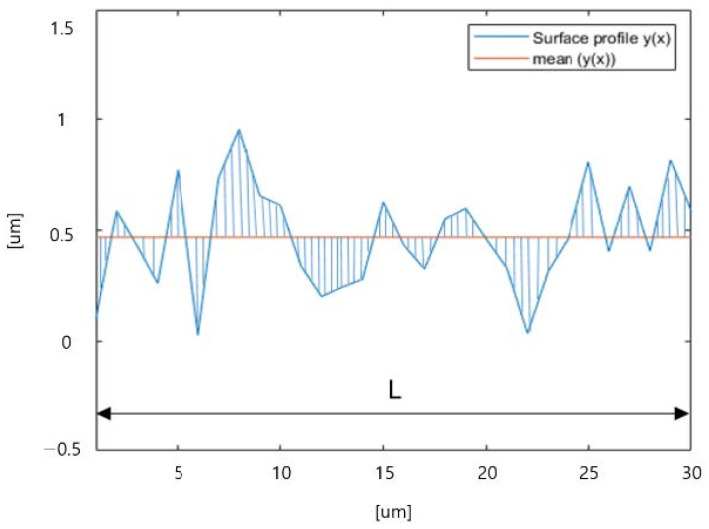
Calculation of surface roughness using surface profile (Ra) [11] (an open-access article (Sensors, MDPI) distributed under the terms of the Creative Commons CC-BY license).

Various methods can be employed to measure surface roughness, depending on different definitions [11,118]. In additive manufacturing research, the Ra parameter is commonly used to measure surface roughness due to its simplicity, though it lacks sensitivity to wavelength variations. As depicted in Figure 3, the shaded area is summed and divided by the length (L) to determine the surface roughness. Studies by Li et al. [119] show that the peak-to-valley distance parameter (*R_z_*) outperforms *R_q_* and *R_a_* in correlating with the tactile and visual assessments of surface roughness. However, relying solely on *R_z_* may not fully encompass the impact of appearance factors such as texture and color on human perception and surface quality. Extracting roughness profiles lacks reproducibility due to instrument dependency and operational factors, as reported in studies [21,120]. While 2D stylus measurements remain popular, non-contact 3D optical profilometry and X-ray CT scanning (ISO 25178-2:2021 [117]) are gaining traction for providing comprehensive information without surface damage.

**Table 3:** Surface roughness metrics based on linear measurement [112,116].

Parameter	Description	Equation	
*R_a_* (roughness average)	The arithmetic average of the absolute values of the roughness profile *	$R_{a}=\frac{1}{l_{e}}\int_{0}^{l_{e}}\left|z\left(x\right)dx\right|$	Equation (1)
*R_q_*	Root Mean Squared of measured microscopic peaks and valleys	$R_{q}=\sqrt{\frac{1}{l_{e}}\int_{0}^{l_{e}}\left|z^{2}\left(x\right)dx\right|}$	Equation (2)
*R_t_* (total height of profile)	The vertical distance between the maximum profile peak height and the maximum profile valley depth along the evaluation length	*Rt* = *max*(*z*(*x*)) – *min*(*z*(*x*))	Equation (3)
*R_sk_* (skewness)	Positive skewness indicates that the surface is made up of peaks and asperities, whereas negative Rsk refers to dominant valleys on the surface	$R_{sk}=\frac{1}{R_{q}^{3}}\frac{1}{l_{e}}\int_{0}^{l_{e}}z^{3}\left(x\right)dx$	Equation (4)
*R_ku_* (kurtosis)	A measure of the sharpness of profile peaks	$R_{ku}=\frac{1}{R_{q}^{4}}\frac{1}{l_{e}}\int_{0}^{l_{e}}z^{4}\left(x\right)dx$	Equation (5)
*RzDIN*	The average distance of peaks to valleys (German Standard)	$R_{zJIS}=\frac{1}{s}\sum_{I=1}^{s}R_{ti}$	Equation (6)
*R_z_JIS*	The average distance of peaks to valleys (Japanese Standard)	$R_{zJIS}=\frac{1}{5}\sum_{I=1}^{5}R_{ti}$	Equation (7)
*η*	Asperity-peak density *	$ɳ=\frac{\frac{m_{4}}{m_{2}}}{6\pi \sqrt{3}}$	Equation (8)
ρ	Asperity-peak radius	$\rho =0.375\sqrt{\frac{\pi }{m_{4}}}$	Equation (9)
σ_s_	The standard deviation of asperity-peak heights *	$\sigma_{s}=\sqrt{1-\frac{0.8968}{\alpha }}\sqrt{m_{0}}$	Equation (10)

* $X=\left\{x\in R,\;0\;\leq \;x\;\leq \;l_{e}\right\},\;m_{0}=AVG\left(Z^{2}\right)$, $m_{2}=AVG\left({\left(\frac{dZ}{dx}\right)}^{2}\right)$, $m_{4}=AVG({\left(\frac{d^{2}Z}{dx^{2}}\right)}^{2}$, $\propto =\frac{m_{o}m_{4}}{m_{2}^{2}}$.

**Table 4:** Area roughness parameters [21,112,121].

Parameter	Description	Equation	
*S_a_*	Deviations in the height of the surface points concerning the Mean Reference Plane of the measurement area (A)	$Sa=\frac{1}{A}\iint A\left|Z\left(x,y\right)\right|$	Equation (11)
*S_z_*	Sum of the largest peak height value and the largest pit depth value within the defined area	*Sz* = *max*(*z*(*x*, *y*)) + *min*(*z*(*x*, *y*))	Equation (12)
*S_q_*	Root mean square surface height	$S_{q}=\sqrt{\frac{1}{A}\iint A^{z^{2}}\left(x,y\right)dxdy}$	Equation (13)
*S_sk_*	The skewness of the surface	$S_{sk}=\frac{1}{s_{q}^{3}}\frac{1}{A}\iint A^{z^{3}}\left(x,y\right)dxdy$	Equation (14)
*S_ku_*	The kurtosis of the surface	$S_{ku}=\frac{1}{s_{q}^{4}}\frac{1}{A}\iint A^{z^{4}}\left(x,y\right)dxdy$	Equation (15)
*RRP*	The reduction in surface roughness	$RRP=\frac{S_{a}^{i}-S_{a}^{f}}{S_{a}^{i}}$	Equation (16)

#### 3.2.2. AI-Based Prediction of Surface Roughness

**Figure 4:**
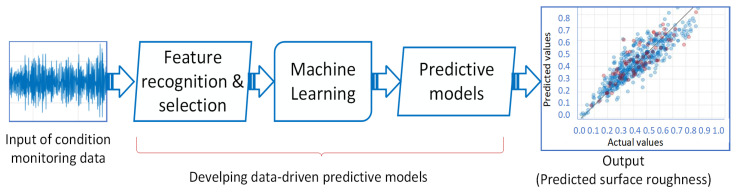
Data-driven predictive modeling.

Figure 4 illustrates the general approach to develop data-driven predictive models that can be used for surface roughness estimation. It illustrates the presence of input, which comprises condition monitoring data, along with predictive modeling, ultimately resulting in the output of surface roughness. These input data, either independently or in combination, are employed to train machine learning models for predicting surface roughness in additively manufactured components. It is crucial to emphasize that both the quality and quantity of the data significantly impact the accuracy and reliability of the predictions. Therefore, the careful collection and processing of data are vital to prevent any potential biases or errors.

The prediction of surface roughness for additively manufactured components is a fundamental application of machine learning, where data-driven modeling is used to predict the surface roughness of additively manufactured components [122]. There are several types of data that can be utilized for this prediction. Commonly used data types include:

Process parameters: These include data related to the additive manufacturing process. In LPBF, for example, parameters such as laser power, scan speed, layer thickness, and hatch spacing [123] have been used for predicting surface roughness. In FDM, parameters such as the feed rate to flow rate, layer thickness, and extruder temperature have been utilized [17]. These parameters can directly impact the surface roughness of the final product.

Material properties: These include data related to the material used in the additive manufacturing process, such as powder size, particle shape, and chemical composition, especially in methods such as L-PBF for metal AM. Material properties can also have a significant impact on surface roughness [124].

Geometrical features: These include data related to the geometry of the final product, such as the angle and direction of the surface, the size and shape of the features, and the number of layers. Geometrical features can influence the surface roughness by affecting the thermal and mechanical properties of the material. For example, in FDM, building orientation has been employed to predict surface quality [125]. In the case of EBM, on the other hand, printed components, the sloping angle and surface orientation have been used to predict surface roughness [126].

Environmental conditions: These include data related to the ambient conditions during the additive manufacturing process, such as temperature, humidity, and pressure. These conditions can affect the material properties and, in turn, the surface roughness of the final product [72].

Surface texture data: These types of data involve measuring the surface roughness of an additively manufactured component using specialized instruments such as profilometers or surface roughness testers. The surface texture data can be used as a training dataset for machine learning models to predict surface roughness based on process parameters, material properties, geometrical features, and environmental conditions [34,127].

Vibration data: Vibration data play a crucial role in predicting surface roughness for additively manufactured components. By capturing and analyzing the vibrational characteristics during the additive manufacturing process, valuable insights can be gained regarding the quality and surface roughness of the manufactured components. Vibration data can help identify any anomalies, such as excessive vibrations or oscillations that may affect the surface roughness. In the FDM-based 3D printing process, for instance, vibration data were integrated to monitor the vibration of the extruder and the table [67]. Vibration data can also be integrated into the machine learning models to enhance the accuracy of surface roughness predictions and further optimize the additive manufacturing process.

## 4. AI-Based Surface Roughness Prediction Methods for Additively Manufactured Parts

In this section, we explore the literature of traditional and deep learning and machine learning algorithms. These incredible algorithms have been used to uncover the secrets of predicting surface roughness in components made through additive manufacturing.

### 4.1. Traditional Machine Learning Approaches

It should be emphasized that conventional machine learning techniques offer a strong basis for surface roughness prediction. However, they might face challenges in capturing intricate non-linear connections or managing data with high dimensions. The application of deep learning techniques has gained prominence in recent years due to their ability to handle these challenges more effectively. The common traditional machine learning algorithms that can be applied for the surface roughness prediction of additively manufactured components are Linear Regression, Support Vector Machine, Random Forests, Gradient Boosting Algorithms, and Artificial Neural Networks.

#### 4.1.1. Support Vector Machines

A surface roughness prediction in the context of additive manufacturing can be facilitated through various supervised learning algorithms. One such algorithm is SVM, which aims to find an optimal hyperplane for data classification. SVM is widely used due to its ability to handle both linear and nonlinear data, as well as its high accuracy [128].

Singh et al. [129] applied SVM techniques to model the Wire Electrical Discharge Machining (WEDM) process of AA6063 for armor applications. They considered four input variables: pulse-on-time (Pon), pulse-off-time (Poff), servo-voltage (V S), and peak-current (IP), with surface roughness as the response parameter. By employing a 3k full factorial design for the experimental runs, the developed model demonstrated its predictive capability and suitability for smart manufacturing. The surfaces of the machined components were further evaluated using SEM analysis.

Joshi et al. [130] explored the applications of supervised learning algorithms, specifically Support Vector Machines and Random Forests, for the surface roughness prediction of FDM-printed parts. Support Vector Machines provide high accuracy in data classification and were used to determine whether the final parts met the desired surface roughness specifications. Random Forests, an ensemble of decision trees, were employed for classification and regression tasks. The study reviewed the implementation of these algorithms and analyzed their applications in additive manufacturing.

Mishra et al. [131] focused on predicting the surface roughness of FDM-printed polylactic acid (PLA) specimens using various supervised machine learning regression-based algorithms. The study utilized explainable AI techniques to enhance the interpretability of the machine learning models. Algorithms such as Support Vector Regression, Random Forest, XGBoost, AdaBoost, CatBoost, the Decision Tree, the Extra Tree Regressor, the Explainable Boosting Model (EBM), and the Gradient Boosting Regressor were employed. The experimental results showed that the XGBoost algorithm achieved the highest coefficient of the determination value (0.9634), indicating its superior accuracy in predicting surface roughness compared to other algorithms. The study also provided a comparative analysis of algorithm performance and insights derived from explainable AI techniques.

#### 4.1.2. Random Forests

Random forests are a type of ensemble model that combines multiple decision trees. Each tree is trained on a different subset of data and features, and the final prediction is obtained through a voting or averaging process. The strength of random forests lies in their ability to handle complex relationships and interactions among input parameters, making them well-suited for surface roughness prediction [132].

Li et al. [17] utilized a random forest (RF) algorithm as part of an ensemble learning-based approach for surface roughness prediction in Fused Filament Fabrication (FFF) processes. The authors incorporated multiple sensors to gather real-time condition monitoring data and extracted a set of features from the raw sensor-based signals in both the time and frequency domains. To enhance computational efficiency and mitigate overfitting, the researchers employed RF to select a subset of 40 features based on their importance. The ensemble learning algorithm in this study combined six different machine learning algorithms, including RF, AdaBoost, CART, SVR, RR, and the RVFL network. The experimental results demonstrated the predictive models’ capability to accurately predict the surface roughness of 3D-printed specimens. The ensemble model outperformed the individual base learners, as evidenced by the lower Root Mean Square Error (RMSE) and Relative Error (RE). The authors concluded that this ensemble learning-based approach, incorporating RF as a feature selection method, shows promise for predicting the surface roughness of additively manufactured components in other processes such as selective laser sintering and electron beam melting.

In their article, Wu et al. [72] addressed the need for real-time process monitoring and predictive modeling in additive manufacturing, with a specific focus on surface roughness prediction in Fused Deposition Modeling (FDM). While existing research has predominantly emphasized physics-based approaches, this study presents a novel data-driven approach using random forests for surface roughness prediction. To achieve this, a real-time monitoring system is developed, integrating multiple sensors to monitor the health condition of a 3D printer and the FDM processes. The RF algorithm is employed to construct a predictive model based on the collected sensor data. The experimental results demonstrate the high accuracy of the predictive model in estimating the surface roughness of printed parts.

Wu et al. [122] utilized random forests to train a model for predicting the surface roughness of parts produced by the FDM process. The study employed a feature-level fusion process to extract features from multiple sensor data, including the temperature of the table and extruder, vibrations of the table and extruder, etc., and combined them into a single feature vector. This vector served as the input to the model which used regression trees to predict the surface roughness value. The study also generated models with individual sensor inputs; however, sensor fusion proved to be a more accurate method, achieving the lowest cross-validation error of 5.91%.

Machine learning algorithms, including random forests, support vector regression, ridge regression (RR), and a least absolute shrinkage and selection operator (LASSO), were employed by Wu et al. [72] to accurately predict surface roughness in manufacturing parts.

#### 4.1.3. K-Nearest Neighbors

K-nearest neighbors (KNN) is a non-parametric algorithm that utilizes proximity to the nearest neighbors to classify or predict data points. In the context of surface roughness prediction, KNN can estimate roughness by identifying the nearest neighbors with similar input parameter values [133].

Kumar and Jain [34] focused on employing the KNN machine learning algorithm for surface roughness prediction. The authors generated training data for the KNN algorithm by depositing multi-layer single-track depositions, resulting in wall-like structures, using Stellite-6 as the additive manufacturing material in both powder and wire forms. Their findings revealed that surface roughness increases with a higher power supply to the micro-plasma and AM material feed rate, while it decreases with an increase in the traverse speed of the deposition head for both powder and wire forms of the AM material. Additionally, the surface roughness of the walls produced with the powder form of the AM material (ranging from 118 to 149 μm) was found to be smaller than that obtained with the wire form (ranging from 195 to 227 μm). The prediction error of surface roughness using the KNN algorithm ranged from −6.2% to 2.8% for the powder form and −5.8% to 2.3% for the wire form of the AM material. These results demonstrate the capability of the KNN algorithm in accurately predicting surface roughness in the μ-PTAMAM process. Furthermore, the authors highlighted that increasing the number of training datasets can further reduce the prediction error of the KNN algorithm.

In the paper [134], the focus is on accurately predicting the surface finish of fused deposition modeling parts using prediction models. Previous models have successfully established a mapping relationship between printing parameters and surface roughness, though they are limited in their ability to handle multi-factor and multi-category predictions and imbalanced data. To address these challenges, a new prediction method called APSO-KNN (adaptive particle swarm optimization and K-nearest neighbor) is proposed. The method considers seven input variables: nozzle diameter, layer thickness, number of perimeters, flow rate, print speed, nozzle temperature, and build orientation. An L27 Taguchi experimental design is utilized to determine the printing values for each specimen. The model is trained and validated using experimental data from 27 printed specimens. The results demonstrate that the proposed method achieves a minimum classification error of 0.01 after two iterations, with a maximum accuracy of 99.0% and high training efficiency. This method successfully meets the requirements for predicting the surface finish in FDM parts with multiple factors and categories, while also addressing imbalanced data. The high accuracy attained by the model indicates its potential for predicting the surface finish and its applicability in actual industrial manufacturing processes.

#### 4.1.4. Artificial Neural Networks (ANN)

ANNs are fascinating machine learning algorithms inspired by the intricate structure and functionality of the human brain. Composed of interconnected nodes called “neurons”, ANNs possess the remarkable ability to process and transmit information. These networks find applications in a wide range of tasks, including pattern recognition, image and speech recognition, prediction, and classification [135].

One captivating application of ANNs lies in the prediction of surface roughness for additively manufactured components [136]. Additive manufacturing, a process that builds 3D objects layer by layer using computer models, presents a challenge in controlling the surface roughness of printed objects—a crucial factor affecting their mechanical properties and overall performance. To address this challenge, ANNs can be trained on datasets comprising input variables and corresponding surface roughness measurements. These input variables encompass factors such as the type of printing material, layer thickness, printing speed, and printing temperature. As ANNs learn from the data, they identify patterns and make predictions based on new inputs. Once trained, ANNs can accurately predict the surface roughness of new additively manufactured components based on their input parameters. This predictive capability empowers manufacturers to optimize their printing processes and enhance the quality of their printed parts [137].

ANN have been widely utilized by researchers to predict the surface roughness of additively manufactured components. In a remarkable study conducted by Wafa and Abdulshahed [138], they employed an ANN approach to accurately predict the surface roughness of FDM-printed components. The ANN model, built with a small number of neurons in the MATLAB environment, exhibited exceptional agreement with the experimental data, achieving an average error value of only 8%. Furthermore, the researchers compared their proposed ANN model to a regression-based approach and found that the ANN model outperformed the statistical method in terms of accuracy.

In another intriguing work, Lakshmi and Arumaikkannu [139] suggested a non-contact method to estimate surface roughness in SLM-customized implants using an ANN. The developed ANN was trained on scan data from a femur bone and achieved an impressive prediction accuracy of 97.2%.

Vahabli and Rahmait [140] made significant advancements by developing a robust model for estimating the surface roughness distribution in FDM parts. They used empirical data and optimized ANN, incorporating particle swarm optimization (PSO) and imperialist competitive algorithm (ICA) techniques. The comprehensive validation of their proposed methodology showcased a superior performance across various conditions and complex shapes, effectively filling a crucial gap in the literature.

The work of Mahapatra and Sood [141] focused on the relationship between input factors and surface roughness using ANN. They applied the Levenberg–Marquardt algorithm to train the neural network, revealing valuable insights regarding the impact of factors of FDM such as raster angle, raster width, layer thickness, orientation, and air gap on surface roughness. In another investigation, Vahabli and Rahmati [142] utilized the radial basis function neural network (RBFNN) to estimate surface roughness. By optimizing the effective parameters using the imperialist competitive algorithm, they achieved improved prediction accuracy compared to using RBFNN alone for FDM parts.

Jiang et al. [143] investigated the response of the FDM process and printable bridge length (PBL) in test specimens. Their study utilized the BP neural network with Bayesian Regularization as the training algorithm, leading to accurate predictions of a test specimen’s PBL. Barrios and Romero [144] deployed decision-tree methods, such as J48, random forest, and random tree, to predict the surface roughness of FDM parts. Through a well-designed dataset and utilizing the Taguchi design and response surface methodology, they successfully screened and optimized surface roughness.

Plaza et al. [145] conducted a study on the effect of build orientation, feed rate, and layer thickness on the surface roughness of workpieces fabricated from PLA filament. Their factorial design method revealed that the layer thickness significantly impacted surface roughness, while the feed rate had no significant effect. They also employed an ANN, utilizing feedforward BP with the Levenberg–Marquardt training algorithm, to predict the surface roughness of FDM printed accurately.

Pfleging [146] aimed to predict and optimize the surface roughness and heat-affected zone depths. The data obtained were utilized to create linear regression and artificial neural network models for each variable. Subsequently, the models with the best performance were employed in a multiobjective genetic algorithm optimization to determine optimal parameter combinations. Through this approach, the research successfully identified an acceptable range of values for the specified input parameters of LPBF (laser power, focal offset, axial feed rate, number of repetitions, and scanning speed) that produced satisfactory values of Ra and HAZ simultaneously. In the pursuit of optimizing the tensile strength, surface roughness, and build time of fabricated work pieces, Yang et al. [48] employed the central composite design (CCD) method. Their study emphasized the significant impact of the nozzle diameter, filling velocity, and layer thickness on surface roughness.

Kandananond [147] utilized the Box–Behnken design to optimize the surface roughness of workpieces fabricated from ABS filament. The study successfully recommended optimal settings for input factors such as nozzle temperature, bed temperature, and printing speed, effectively minimizing surface roughness. The comparison study between the response surface method (RSM) and machine learning methods, including ANN and fuzzy inference system (FIS), provided valuable insights into the relationships between the inputs and outputs of the FDM system.

Lastly, Ulkir et al. [148] conducted a study to optimize the prediction model for minimizing surface roughness in FDM samples. They used a combination of a Cascade-Forward Neural Network (CFNN) and a genetic algorithm to determine the optimal combination of input parameters, yielding impressive results.

#### 4.1.5. Others Machine Learnings

There are other machine learning algorithms used for surface roughness prediction. For instance, a machine learning method based on Gaussian Process Regression was proposed to establish a model relating the WAAM process parameters to the top surface roughness. To measure the top surface roughness of a manufactured part, a 3D laser measurement system was developed. The experimental datasets were collected and subsequently divided into training and testing datasets. Using the training datasets, a top surface roughness model was constructed and then verified using the testing datasets. The experimental results demonstrate that the proposed method achieves a surface roughness prediction accuracy of less than 50 µm [149].

Li et al. [17] introduced the ensemble learning algorithm to determine predictive models for surface roughness. Their approach involved training the model using different learning algorithms, including random forests, AdaBoost, classification and regression trees (CART), SVR, RR, and random vector functional link (RVFL) networks. The experimental results demonstrated the effectiveness of this ensemble learning-based approach, particularly in accurately predicting the surface roughness of fused filament fabrication (FFF)-manufactured components.

Mishra and Jatti [92] conducted a comprehensive comparison of three quantum algorithms—Quantum Neural Network (QNN), Quantum Forest (Q-Forest), and Variational Quantum Classifier (VQC)—adapted for regression to predict surface roughness in additively manufactured components. Through the evaluation of the Mean Squared Error (MSE), Mean Absolute Error (MAE), and Explained Variance Score (EVS), the study reveals that the Q-Forest algorithm outperforms the others, achieving an MSE of 56.905, MAE of 7.479, and an EVS of 0.2957. In contrast, the QNN algorithm exhibits a higher MSE and MAE, along with a negative EVS, suggesting its limited suitability for surface roughness prediction. The VQC adapted for regression also demonstrates an inferior performance compared to the Q-Forest algorithm.

### 4.2. Deep Learning Approaches

Machine learning techniques are considered a weak AI type, which means they are not entirely autonomous and require some level of guidance, such as adjusting hyperparameters. Deep learning (DL) methods were developed to push beyond the limitations of traditional machine learning, which are also aimed to emulate certain aspects of human cognition more closely. Consequently, they have proved to have a superior performance in terms of accuracy and speed compared to other machine learning algorithms, without the need for significant manual intervention from programmers. Deep learning is a specific subset of machine learning that operates by processing inputs through a biologically inspired ANN architecture. Over time, it has become evident that neural networks outperform many other algorithms in accuracy and speed due to their powerful ability to extract relevant information from vast amounts of data [150].

**Figure 5:**
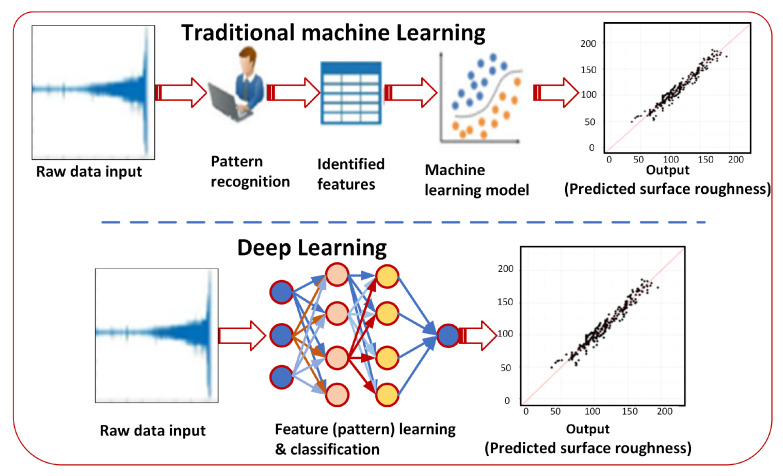
Traditional machine learning vs deep learning for surface roughness prediction.

As can be observed from Figure 5, the main difference between deep learning and traditional machine learning lies in the architecture and representation of data. In traditional machine learning, feature engineering is a separate step where domain experts manually select and engineer relevant features from the raw data. While traditional methods may be more interpretable, they heavily rely on domain knowledge and might struggle to capture intricate relationships or patterns in the data without comprehensive feature engineering. Moreover, the feature engineering process can be time-consuming and subjective. However, in deep learning, feature extraction and prediction are performed in an end-to-end manner using neural networks. Raw data are directly fed into the network, and the model automatically learns hierarchical representations and complex features, eliminating the need for manual feature engineering. The neural network’s layers capture abstract patterns and relationships within the data, enabling it to predict surface roughness directly from the raw input, making the process more efficient and accurate with a large amount of labeled data. However, the black-box nature of deep learning models may limit interpretability [151].

**Figure 6:**
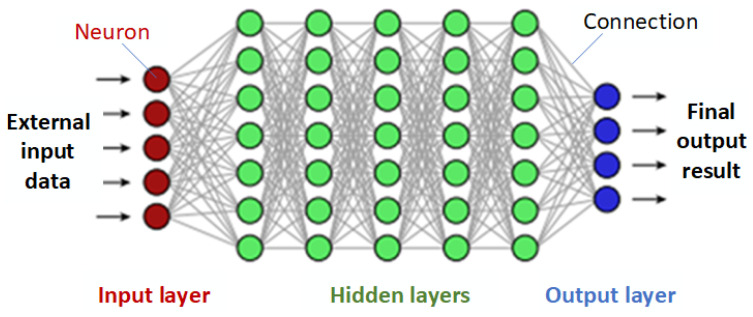
Schematic representation of a deep neural network, a type of artificial neural network featuring several hidden layers of neurons between the input and output layers.

Deep learning possesses remarkable capabilities for modeling and handling highly intricate non-linear relationships. Numerous variants of deep learning techniques are available, such as convolutional neural networks (CNNs), recurrent neural networks (RNNs), artificial neural networks (ANNs), and deep neural networks (DNNs). These neural networks consist of artificial neurons organized in multiple layers, where each layer communicates only with the immediately preceding and following layers (as shown in Figure 6). Information flows through the neural network from the input layer (which receives external data) to the output layer (which produces results), passing through several hidden layers in between. The number of hidden layers depends on the complexity of the problem being solved. In each hidden layer, neurons receive input signals from other neurons, process them by combining the input with their internal state, and produce output signals. Neurons are interconnected where the output of one serves as input to another, and each neuron may have multiple input and output connections. Specific weights are then assigned, forming the overall layer of the neural network. The learning process involves adjusting the network’s parameters, specifically the weights of the connections, by minimizing observed errors in the network’s output. Due to its versatile and self-adapting architecture, deep learning reduces the need for extensive feature engineering and can effectively identify and address challenges that may be challenging to detect using other techniques. However, training artificial neural networks demands a substantial amount of data for accurate learning and is computationally expensive due to the vast number (millions or more) of parameters that require optimization during the training process.

Deep learning methods commonly used for surface roughness prediction of additively manufactured components include conventional neural networks, deep neural networks, generative adversarial networks, auto encoders, and deep reinforcement learning. These methods are briefly explained below.

#### 4.2.1. Convolutional Neural Networks

Convolutional Neural Networks (CNNs) are widely used in image recognition and classification tasks, particularly in the analysis of surface topography scans and visual representations of additively manufactured components. These networks utilize convolutional layers to extract hierarchical features from input data, allowing them to capture intricate patterns and textures related to surface roughness. Researchers have recently employed CNNs for predicting the surface roughness of additively manufactured components [152].

In a recent study [153], a deep learning CNN model was utilized to predict the surface roughness of additively manufactured components. The study proposed a combined approach of CNN classification and electrical discharge-assisted post-processing to enhance the surface quality of these components. By categorizing the surface, the depth and number of polishing passes were determined. The study revealed that polishing under a low-energy regime outperformed high-energy regimes, resulting in a significant 74% improvement in surface finish. Additionally, lower-energy polishing reduced the occurrence of short-circuit discharges and elemental migration. The CNN model demonstrated 96% accuracy in predicting the surface condition through a five-fold cross-validation. Furthermore, the proposed approach substantially improved the surface finish from 97.3 to 12.62 μm.

In another work [154], researchers demonstrated that CNNs could directly predict the energy required to deform gyroid and octet truss metamaterials using only optical images. By exploiting the tiled nature of engineered lattices, the small dataset was augmented by subdividing the original image into smaller sub-images. During testing, the predictions from these sub-images were combined to estimate the deformation work of the entire lattice. This approach provided a rapid and cost-effective screening tool for predicting properties of 3D-printed lattices, surpassing mere inspection, and accurately estimating performance metrics, rather than just detecting defects.

In a paper [155], an automated quality grading system for the additive manufacturing process was proposed, employing a deep CNN model. The CNN model was trained offline using images of internal and surface defects in the layer-by-layer deposition of materials and tested online to detect and classify failures in the AM process at different extruder speeds and temperatures. The model exhibited an accuracy of 94% and specificity of 96%, with an F-score, sensitivity, and precision measures above 75% for classifying the quality of the printing process into five grades in real-time. The proposed online model introduced automated, consistent, and non-contact quality control, eliminating the need for the manual inspection of completed parts. The quality monitoring signal could also be utilized by the machine to suggest real-time adjustments in parameters and remedial actions. This predictive model serves as a proof-of-concept for any AM machine, facilitating the production of reliable parts with fewer quality issues and minimizing wastage of time and materials.

Machine learning can be leveraged to automate common or time-consuming engineering tasks using existing data. For example, design repositories can be employed to train deep learning algorithms to assess component manufacturability. However, methods for determining the suitability of design repositories for machine learning are lacking [156]. A study investigated this matter by employing “artificial” design repositories to assess the impact of altering dataset properties on the precision and generalizability of neural networks trained on the data. A 3D convolutional neural network was employed to estimate manufacturing metrics directly from voxel-based component geometries, focusing on additive manufacturing as a case study. The study examined three build metrics: part mass, support material mass, and build time. The results indicated that training on design repositories with a less standardized orientation and position resulted in more accurate neural networks. Furthermore, orientation-dependent metrics were more challenging to estimate than orientation-independent metrics. The convolutional neural network outperformed the baseline linear regression model for all build metrics. 

Furthermore, a machine learning integrated design for the AM framework was proposed, utilizing ML’s ability to learn complex relationships between design and performance spaces. A case study demonstrated ML’s effectiveness in designing a customized ankle brace with a tunable mechanical performance and tailored stiffness [157].

#### 4.2.2. Deep Neural Networks

A deep neural network (DNN) is an ANN having multiple layers between the input and output layers. A DNN consists of neurons, synapses, biases, weights, and functions. Deep neural networks have demonstrated discriminative and representative learning capabilities across various applications in recent years [158,159].

So et al. [11] developed a method to improve the quality of additively manufactured products by predicting surface roughness using data analysis techniques, including data pre-processing and DNNs combined with sensor data. The study focused on enhancing the surface roughness of the stacked wall, which is a crucial quality indicator affecting product life and structural performance. By applying data pre-processing and DNNs with sensor data, the study proposed a methodology to predict surface roughness based on process parameters. The effectiveness of the proposed methodology was validated using field data from wire + arc additive manufacturing, resulting in a mean absolute percentage error (MAPE) of 1.93%.

In the prediction of surface roughness for an AlSi10Mg aluminum alloy fabricated through laser powder bed fusion (LPBF), a deep learning framework was developed [160]. The framework involved fabricating specimens, measuring surface topography, extracting and streamlining roughness and LPBF processing data, and developing a deep neural network model. The inputs to the model were the AM process parameters, and the output was the surface profile height measurements. The deep learning framework successfully predicted the surface topography and roughness parameters for all printed specimens, with measurements well within 5% of experimental error.

In the study on selective laser melting, a deep neural network was applied to classify melt-pool images based on laser power labels [161]. The neural network demonstrated effective classification, even with melt-pool images featuring blurred edges. The classification model showed a failure rate of under 1.1% for 13,200 test images, proving its effectiveness in monitoring melt-pool images and identifying potential defects.

Post-processing methods, such as shot peening (SP), severe vibratory peening (SVP), and laser shock peening (LSP), are commonly employed to address surface imperfections and bulk defects in additive manufactured materials. In a previous study, fracture surfaces of failed samples were analyzed, and experimental data were elaborated using a machine learning-based approach [162]. The study developed a six-layer deep neural network and utilized a stacked auto-encoder (SAE) for pre-training the dataset. The ML-based model achieved prediction accuracies of over 0.96 and revealed correlations between the residual stress, hardness, surface roughness, crack initiation site depth, and fatigue life of the post-treated samples.

#### 4.2.3. Generative Adversarial Networks

Generative Adversarial Networks (GANs) have shown promise in predicting the surface roughness of additively manufactured components by generating synthetic profiles that closely resemble real-world data. GANs consist of a generator network and a discriminator network that compete against each other, enabling them to learn the underlying distribution of surface roughness and aid in prediction tasks [163].

In a recent paper [164], a novel image processing method was proposed to enhance the quality of thermal images for feature extraction in the Directed Energy Deposition (DED)-based additive manufacturing process. The method utilized an Improved Enhanced Generative Adversarial Network (IEGAN) with a modified objective function. A penalty term was introduced to enhance the contrast ratio of the reconstructed thermal images. The effectiveness of the proposed IEGAN was demonstrated by comparing the contrast ratio with that of the original GAN. The IEGAN successfully extracted the shape of the melt pool, contributing to process monitoring in additive manufacturing.

To address the challenge of controlling porosity in metal additive manufacturing, a framework combining Generative Adversarial Networks and Mallat Scattering Transform-based autocorrelation methods was introduced [165]. This framework deconstructed the generation problem and generated individual pore geometries and surface roughness. These generated components were then stochastically reconstructed to form realizations of a porous printed part. Statistical and dimensional metrics were used to compare the generated parts with existing experimental porosity distributions.

In laser powder bed fusion (L-PBF), predicting the morphology of the melt pool is crucial for controlling the final build quality. Machine learning techniques were leveraged to predict both the quantitative attributes (size) and qualitative attributes (shape) of the melt pool [166]. An LSTM network was used to predict the area of the melt pool with high accuracy, while a Melt Pool Generative Adversarial Network (MP-GAN) synthesized images of the melt pool with high structural similarity scores. These predictions enabled the real-time monitoring and control of the melt pool for consistently better quality in L-PBF.

A novel method utilizing artificial intelligence was proposed to generate the virtual surface morphology of Ti-6Al-4V parts based on given process parameters [167]. Conditional generative adversarial networks were optimized to develop a high-resolution surface morphology image generation system. The virtual surface closely matched experimental cases, exhibiting reduced textured microstructural behavior on the surface and decreased anisotropy in the columnar structure. This AI-guided virtual surface morphology approach can help obtain high-quality parts more cost-effectively.

In the context of quality assurance in advanced manufacturing processes such as additive manufacturing, imbalanced training data can hinder the detection of abnormal states. To address this issue, a data augmentation method based on a Generative Adversarial Network was proposed [168]. By jointly optimizing a standard GAN and a classifier, high-quality generated samples were used to provide a balanced training set, improving the performance of abnormal state detection in polymer and metal additive manufacturing processes.

#### 4.2.4. Autoencoders

Autoencoders are a type of unsupervised learning model used for dimensionality reduction and feature extraction. They encode and decode input data to learn compact representations of surface roughness profiles. These representations can be used in traditional machine learning models or for visualization purposes [169].

In the context of additive manufacturing, post-processing methods are commonly employed to address surface imperfections and bulk defects. Previous studies analyzed the effects of different peening-based treatments on the fatigue performance of laser powder bed fusion AlSi1Mg samples. The fracture surfaces of failed samples were further analyzed, and machine learning-based approaches were utilized to identify correlations between the residual stress, hardness, surface roughness, depth of the crack initiation site, and fatigue life of the post-treated samples. A deep neural network (DNN) and stacked autoencoder (SAE) were used to develop a machine learning model, with the SAE providing accurate predicted results. Parametric analyses and sensitivity analyses were performed to assess the importance of each input factor. The results showed that enhancing surface hardening, inducing higher compressive residual stresses, and reducing surface roughness led to deeper crack initiation sites and improved fatigue life [162].

Additive manufacturing is increasingly used in quality-critical applications, such as aerospace and healthcare, for fabricating complex geometries with novel materials. However, the layer-wise surface quality in additive manufacturing can be relatively poor, affecting the properties and functionality of the products. Surface morphology studies have demonstrated the significant impact of AM machine parameters on the resulting surface. Analyzing and correlating morphology features with machine parameters pose challenges due to the highly nonlinear nature of surface profiles and the presence of outliers and missing regions. To address these challenges, a convolutional autoencoder-based approach is applied in this paper to extract informative features from surface profiles. The autoencoder model, with regularization, can effectively discover low-dimensional representations from high-dimensional surface profile data, even in the presence of corruptions. Supervised machine learning is then employed to quantify the correlation between surface morphology and machine parameters. A case study in laser engineered net shaping (LENS) validates the effectiveness of the proposed method, achieving a classification accuracy of 70%, outperforming benchmark methods. Thus, the developed convolutional autoencoder-based approach shows promise for extracting surface morphology features in additive manufacturing [170].

#### 4.2.5. Deep Reinforcement Learning

Deep Reinforcement Learning (DRL) is a combination of deep learning and reinforcement learning techniques. In the context of surface roughness prediction in additive manufacturing, DRL has emerged as a promising approach for optimizing the manufacturing process to achieve desired surface roughness outcomes. By employing DRL agents, which are intelligent systems, optimal control policies can be learned through interaction with the manufacturing environment and feedback on surface roughness performance [171].

This study introduces a DRL framework for deriving a versatile control strategy aimed at minimizing the occurrence of defects. The control policy generated by the DRL framework modifies either the velocity or power of the laser during the melting process. This adjustment ensures the consistency of the melt pool and reduces overheating in the final product. To train and validate the control policy, efficient simulations of the temperature distribution within the powder bed layer are conducted under different laser trajectories [172].

## 5. Discussion

### 5.1. Analysis of AI Techniques Used to Predict Surface Roughness

The presented studies demonstrate a diverse range of AI techniques employed for surface roughness prediction in additively manufactured components. Each study offers unique insights into the efficacy of different methodologies, shedding light on the advantages and limitations of AI-driven approaches in this domain. Table 5 lists a summary of some selected works on the data used as the input, the models developed, and the results obtained from the surface roughness prediction models.

#### 5.1.1. Data Input Variability

The variety of input data used across these studies highlights the adaptability of AI techniques to different additive manufacturing processes. Mishra et al. [131] utilized a comprehensive set of FDM parameters, while Xia et al. [173] focused on process parameters specific to the WAAM process. The success of these studies underscores the potential for AI models to handle various input types, capturing intricate relationships between parameters and surface roughness.

#### 5.1.2. Model Performance and Accuracy

The performance metrics across the studies, such as R2, RMSE, and MAE, reflect the success of AI models in capturing complex patterns and providing accurate surface roughness predictions. The notable performance of the XGBoost algorithm in the study by Mishra et al. [131] highlights its suitability for handling a multitude of FDM parameters, showcasing its ability to learn and model complex interactions. Similarly, the GA–ANFIS model introduced by Xia et al. [173] exhibited a superior predictive performance. The use of genetic algorithms to optimize ANFIS parameters contributed to accurate predictions, showcasing the power of hybrid models in enhancing accuracy.

#### 5.1.3. Interpretability vs. Black Box Models

While deep learning techniques, such as ANNs and XGBoost (https://xgboost.ai/), consistently demonstrated high accuracy, they often sacrifice interpretability. In contrast, ensemble methods such as the Random Forest Regressor, as seen in Saxena et al. [91], and the GA–ANFIS model in Xia et al. [173], offer a balance between accuracy and interpretability. This trade-off is significant, particularly in industries, where understanding the underlying factors influencing predictions is crucial for process optimization and decision making.

#### 5.1.4. Application to Diverse Manufacturing Processes

The versatility of AI models is evident in their application to various additive manufacturing processes. Studies such as Huang et al. [134] demonstrate the feasibility of AI techniques in predicting surface roughness for FDM, while Singh et al. [129] applied Support Vector Machine (SVM) techniques to the domain of WEDM. This showcases the potential for AI models to be tailored to different manufacturing processes, enabling their wide-ranging adoption.

#### 5.1.5. Optimizing Manufacturing Processes

The studies collectively underline the potential of AI techniques in optimizing additive manufacturing processes. These models have the capability to predict surface roughness accurately, guiding engineers to adjust parameters for desired outcomes without extensive trial and error. This optimization potential can lead to enhanced manufacturing efficiency, reduced material waste, and improved product quality.

#### 5.1.6. Weakness and Strength of each Developed Model

Each developed model possesses unique strengths and limitations when predicting the surface roughness of additively manufactured components. Considering the diversity of data types, a comparative analysis of the strengths and weaknesses was conducted for both machine learning models and deep learning models employed in surface roughness prediction. The strengths and weaknesses of the machine learning models were presented in Table 6, while those of the deep learning models were outlined in Table 7.

### 5.2. Challenges and Limitations

The literature on the application of AI techniques for surface roughness prediction in additive manufacturing highlights various limitations and challenges that must be addressed to advance this field.

One significant limitation is the scarcity of publicly available datasets containing comprehensive surface roughness measurements. Many studies rely on proprietary or limited experimental data, making it challenging to develop and evaluate AI models. The lack of standardized datasets hampers the ability to compare and benchmark different approaches. Additionally, the limited availability of diverse data restricts the training of AI models in a wide range of additive manufacturing scenarios, limiting their ability to generalize. This scarcity of high-quality datasets poses a challenge in achieving accurate predictions of surface roughness using AI models. Indeed, the scientific community is making continuous efforts to tackle the surface roughness issue in additive manufacturing. For instance, researchers are actively exploring methods such as supervised machine learning regression-based algorithms to predict the surface roughness of additive-manufactured polylactic acid specimens [131]. Additionally, data-driven predictive modeling approaches are being developed to enhance surface roughness prediction in additive manufacturing processes. These research endeavors aim to improve the understanding and control of surface roughness in the context of additive manufacturing, ultimately leading to the better quality and performance of the manufactured parts.

Another challenge lies in feature selection and extraction. While some studies have explored manual feature engineering techniques, these processes can be time-consuming and subjective. Furthermore, relevant feature selection depends heavily on the specific additive manufacturing process and material. Although automated feature learning using deep learning models has gained attention, determining the most informative features from complex datasets remains a challenge. Further research is necessary to develop robust and automated feature selection methods tailored specifically for surface roughness prediction in additive manufacturing.

The generalization of AI models across different additive manufacturing processes and materials poses another significant challenge. Each combination of process and material exhibits unique characteristics that influence surface roughness. AI models trained on specific process–material combinations may not effectively transfer to other scenarios, limiting their applicability. Developing transferable models capable of handling variations in process parameters, material properties, and surface characteristics is crucial for the broader adoption of AI techniques in predicting surface roughness. Additionally, the complexity and variability of additive manufacturing processes make it difficult to develop models that accurately predict surface roughness across different materials and printing parameters. Capturing the complex and nonlinear relationship between process parameters and the resulting surface roughness is crucial. A data-driven predictive modeling approach that utilizes multiple sensors to collect temperature and vibration data has been introduced to improve the surface integrity of additively manufactured parts.

Furthermore, the interpretability and explainability of AI models present challenges in this domain. Many AI models, especially deep learning models, are often perceived as black boxes, making it difficult to understand the reasoning behind their predictions. This lack of interpretability hampers insights into the factors contributing to surface roughness. Research efforts should focus on developing explainable AI models that shed light on the relationships between process parameters, material properties, and surface roughness. Ensuring the transparency and interpretability of these models is crucial for their successful integration into real-world manufacturing processes.

In addition to the aforementioned challenges, training challenges and computational costs also play a significant role. Training AI models for accurate surface roughness prediction demands substantial computational resources and time. The complexity of the underlying data, along with the need for large and diverse datasets, often results in prolonged training periods. Furthermore, finding an optimal balance between model complexity and generalization capacity is a challenge. Models that are overly complex might overfit to training data and perform poorly on unseen data, while overly simplified models might lack the ability to capture intricate relationships in the data.

Considering computational costs is paramount, especially in real-time applications where quick predictions are required. Complex AI models, such as deep neural networks, can demand substantial computational power for both training and inference phases. This can lead to challenges in deployment, especially in resource-constrained environments. Optimization techniques and hardware acceleration solutions are being explored to mitigate these computational challenges.

Data quality and preprocessing complexity present further challenges. Acquiring precise and dependable measurements of surface roughness can be complex due to factors such as measurement equipment accuracy, noise, and variations in measurement techniques. Ensuring consistent data quality across diverse sources is essential for training reliable AI models. Additionally, the process of preprocessing raw data to eliminate outliers, address missing values, and standardize data for training can introduce its own intricacies and potential sources of error.

Moreover, integrating AI techniques into existing manufacturing processes and systems presents implementation challenges. Incorporating AI-based surface roughness prediction models into real-time control systems requires considerations such as computational efficiency, real-time data acquisition, and compatibility with existing manufacturing infrastructure. Addressing the implementation and deployment of AI models in practical manufacturing environments is vital to ensure seamless integration and practical utility.

In conclusion, while AI techniques hold promise for surface roughness prediction in additive manufacturing, several limitations and challenges must be overcome. These include the scarcity of comprehensive and standardized datasets, challenges in feature selection and extraction, limitations in model generalization, a lack of interpretability, and implementation challenges. Addressing these limitations and challenges will pave the way for more accurate, robust, and practical AI-based surface roughness predictions in additive manufacturing.

### 5.3. Future Direction

The existing literature on the application of AI techniques for surface roughness predictions in additive manufacturing suggests several promising future directions that can further advance this field. These directions address the limitations and challenges identified and aim to enhance the accuracy, applicability, and practicality of AI-based surface roughness predictions.

One crucial future direction is the integration of AI techniques with process optimization algorithms. By combining predictive models with closed-loop control systems, real-time adjustments can be made to process parameters during additive manufacturing. This integration enables the dynamic control of surface roughness, leading to improved surface quality control. The optimization algorithms can utilize the predicted surface roughness values to adjust process parameters, ensuring the desired surface roughness is achieved. This integration holds the potential to optimize additive manufacturing processes and improve overall manufacturing efficiency.

The utilization of generative AI models, such as generative adversarial networks (GANs), is another promising future direction. GANs can be employed to generate synthetic surface roughness patterns that resemble real-world variations. These generative models can augment limited datasets, simulate different surface roughness scenarios, and enhance the robustness of AI models. By generating synthetic data, GANs can alleviate the data scarcity issue and improve the generalization capabilities of AI models.

The development of explainable AI models is also critical for the future of surface roughness predictions in additive manufacturing. Explainability is essential for users and stakeholders to understand and trust the predictions made by AI models. Methods for explaining the decision-making process of AI models need to be explored and implemented. This can provide insights into the factors influencing surface roughness predictions and enable users to identify opportunities for process optimization and improvement. Explainable AI models can enhance transparency, trust, and acceptance of AI techniques in the additive manufacturing industry.

Currently, the majority of studies in surface roughness prediction for additively manufactured components primarily concentrate on process parameters. Only a limited number of papers explore alternative data sources such as vibration data, material properties, environmental conditions, and geometric features for predictive modeling. Future research endeavors should explore and integrate these underutilized data types to enhance the accuracy and robustness of surface roughness predictions in additive manufacturing.

Additionally, future research should focus on exploring advanced feature selection techniques specifically tailored for surface roughness prediction in additive manufacturing. The development of automated feature extraction methods that can effectively capture the underlying factors influencing surface roughness would enhance the predictive performance of AI models. Advanced feature selection techniques, such as deep feature learning or genetic algorithms, can help identify the most relevant and informative features from complex datasets.

## 6. Conclusions

This review paper has explored the application of AI for surface roughness predictions of additively manufactured components. The literature review has provided an overview of the recent advancements in machine learning and AI deep learning techniques employed by researchers in this field. The findings demonstrate that AI-based methods have shown promising results in predicting surface roughness, offering benefits such as cost reduction and time savings. However, certain limitations and challenges, including the availability of quality data and model interpretability, need to be addressed. Future research should focus on addressing these challenges, developing more accurate and robust models, and integrating AI methodologies into the additive manufacturing process. Overall, leveraging AI for surface roughness predictions holds great potential for improving the productivity, competitiveness, and consistent product quality of additively manufactured components. Future research should focus on addressing the challenges and refining AI-based approaches to enhance surface quality control in additive manufacturing processes.

## Figures and Tables

**Table 1:** Surface roughness of most additively manufactured techniques.

Name of AM process	Minimum Layer Thickness		Surface Roughness, Ra	
	Values (mm)	Ref.	Values (µm)	Ref.
Stereolithography (SLA)	0.1	[18]	2–10	[19]
Selective Laser Sintering (SLS)	0.02	[20]	5–25	[21]
Fused Deposition Modeling (FDM)	0.05	[22]	0.5–20	[23]
Laminated Object Manufacturing (LOM)	0.10	[24]	0.8–2.5	[25]
Electron Beam Melting (EBM)	0.05	[26]	1–20	[27,28]
Direct Metal Laser Sintering (DMLS)	0.02	[29]	3–12	[30]
Binder Jetting	0.035	[31]	3–13	[32]
Direct Energy Deposition (DED)	0.25	[33]	5.08–227	[34,35]
Laser powder bed fusion (L-PBF)	0.02	[36]	3.5–13.45	[37]
Selective Laser Melting (SLM)	0.02	[38]	30–60	[39]

**Table 2:** Process parameters influencing surface quality of AM manufactured components of most additively manufactured techniques.

AM Technique	Key Process Parameters Influencing Surface Quality	References
FDM/FFF	Layer thickness, Build Orientation, Raster Angle/Raster Orientation, Air Gap, Extrusion temperature, Print Speed, Infill pattern, Infill density/Interior infill, Nozzle diameter, Raster width, Number of contours, Contour width, Contour to contour Air gap	[51,57]
Powered-based fusion	Layer Thickness, Temperature, Laser Power, Beam Radius, Reflection Coefficient at Bed Powder Surface, Material Local Density, Velocity, Conductivity, Dynamic Viscosity	[55,58]
Direct energy deposition	Laser power, Scan speed, Powder feed rate, Travel speed, Layer height, Hatch spacing	[33,56,59]
Vat photopolymerization	Grayscale, Exposure time, Wavelength, Power source, Layer thickness	[60,61]
Binder jetting	Spreader speed, Layer height, Print head speed, Saturation level	[47,62]
PolyJet by Stratasys or MultiJet Printing (MJP) by 3D Systems	Print heads, Hardness range, Layer thickness, Material, Part size, Print resolution	[63]
Sheet lamination	Laser power, different exposure times	[64]

**Table 5:** The data used, the model developed, and the results of some selected surface roughness prediction models.

Authors	AM	Data Used as an Input	The Model Used/Developed	Remarks/Results
Mishra et al. [131]	FDM/FFF	The input parameters of FDM techniques used for the study were layer height, wall thickness, infill density, infill pattern, nozzle temperature, bed temperature, print speed, and fan speed.	Support Vector Regression, Random Forest, XGBoost, AdaBoost, CatBoost, Decision Tree, the Extra Tree Regressor, the Explainable Boosting Model (EBM), and the Gradient Boosting Regressor.	The XGBoost algorithm outperforms the other algorithms with the highest coefficient of determination value of 0.9634. This provides the most accurate predictions for surface roughness compared with other algorithms.
Xia et al. [173]	WAAM	Data from the WAAM process parameters, specifically Welding speed (mm/s), Wire feed speed (m/min), and Overlap ratio were collected using a full factorial design.	ANFIS, ELM, and SVR machine learning models were developed to predict the surface roughness.	The comparison results indicate that GA–ANFIS has superiority in predicting surface roughness. The RMSE, R^2^, MAE, and MAPE for GA–ANFIS were 0.0694, 0.93516, 0.0574, and 14.15%, respectively.
Huang et al. [134]	FDM	Process parameters of nozzle diameter, layer thickness, number of perimeters, flow rate, print speed, nozzle temperature, and build orientation were used.	The model which combines the adaptive particle swarm optimization and K-nearest neighbor (APSO-KNN) algorithms was developed.	The results indicate that the proposed method can achieve a minimum classification error of 0.01 after two iterations, with a maximum accuracy of 99.0%, and high model training efficiency.
Saxena et al. [91]	FDM	Five parameters that influence layer geometries: layer height, infill density, printing speed, and nozzle temperature with a 0-degree raster angle were used.	Support vector machine, linear regression, and two ensemble learning techniques, Xtremegradient boosting and random forest regressor model, were used for prediction of surface roughness.	By applying all machine learning algorithms, random forest regression is the best model, which gives 94.85% accurate results in datasets with a minimum mean squared error of approximately 0.1255 and a maximum r2 score of approximate. 0.9685.
Kandananond [174]	FFF	Bed temperature (80, 85, and 90 °C), printing speed (40, 80, and 120 mm/s), and layer thickness (0.10, 0.25 and 0.40 mm).	Box–Behnken and ANN	The application of ANN to accurately predict surface roughness is also recommended in this study.
Singh et al. [129]	WEDM	Four input variables, namely pulse-on-time (Pon), pulse-off-time (Poff), servo-voltage (VS), and peak-current (IP) were used as data.	Support Vector Machine Technique	The correlation value of the SVM model was 0.9634. The maximum value of roughness, i.e., 6.777 µm, was achieved for Pon = 125 μs, Poff = 60 μs, IP = 30 A, and VS = 40 V; while, the minimum value of roughness, i.e., 2.381 µm, was obtained for Pon = 105 μs, Poff = 40 μs, IP = 150 A, and VS = 60 V. Close to 65% improvement in surface finish value was achieved owing to a suitable setting of machining parameters.
Wafa and Abdulshahed [138]	FDM	Nozzle temperature (°C), layer height (μm), printing speed (mm/s), Nozzle diameter (mm), and Infill density (%) considered as input variables were used.	Artificial Neural Networks (ANN)	Results show that the proposed model has high accuracy in comparison to the statistical approach. Therefore, we can use the ANN model to predict the part surface roughness for 3D printing technology. The results obtained with the proposed ANN model are also superior to the regression-based model.
Wu et al. [72]	FDM	The condition monitoring data include (1) the temperature of the build plate, (2) the temperature of the extruder, (3) the vibration of the build plate, (4) the vibration of the extruder, and (5) the temperature of the deposited material.	The predictive models are trained using random forests (RFs), support vector regression (SVR), ridge regression (RR), and least absolute shrinkage and selection operator (LASSO).	The experimental results have shown that the predictive models trained by the machine learning algorithms on the condition monitoring data predict the surface roughness of additive manufacturing parts with very high accuracy.
Li et al. [17]	FFF	Various types of sensors, such as thermocouples, infrared temperature sensors, and accelerometers, are employed to gather data on temperature and vibration.	The ensemble learning algorithm combined six different machine learning algorithms, including RF, AdaBoost, CART, SVR, RR, and RVFL network, were used.	The experimental results have shown that the predictive models are capable of predicting the surface roughness of the 3D-printed specimens. The performance of the ensemble outperforms the individual base learners based on RMSE and RE.
So et al. [11]	(GMAW)-CMT WAAM	Dynamic process parameters (travel speed, feed rate), Point clouds of bead shape.	Deep Neural Networks (DNN).	Proposed DNN model achieves 98% prediction accuracy, outperforming regression and SVR models. Uses dynamic parameters and bead shape for surface roughness prediction.
Koo et al. [175]	Powder Bed Fusion with Laser Beam (PBF-LB)	Laser power, Scanning speed, Hatching distance, Overhang angle.	Support Vector Regression (SVR), Random Forest (RF), Multilayer Perceptron (MLP).	Machine learning algorithms (SVR, RF, MLP) applied to predict downskin surface roughness in PBF-LB process. Input variables include process parameters and overhang angle. RF model demonstrated most promising results. Average deviations for SVR, RF, and MLP were 13.7%, 4.3%, and 22.5%, respectively, compared to actual measured roughness. RF showed highest prediction accuracy without using sensors.
Kumar and Jain [34]	μ-PTAMAM (Micro-Plasma Transfer Arc Metal Additive Manufacturing)	Power supply, AM material feed rate, Traverse speed.	K-nearest neighbors (KNN).	KNN predicts surface roughness in μ-PTAMAM. Training data from Stellite-6 in powder and wire forms. Powder form has lower roughness (118–149 μm) than wire (195–227 μm). KNN error: −6.2% to 2.8% (powder), −5.8% to 2.3% (wire).

**Table 6:** The strengths and weaknesses of each machine learning algorithm used for surface roughness prediction of additively manufactured components.

**Technique**	**Strengths**	**Weaknesses**
Support Vector Machines (SVM)	- Effective in high-dimensional spaces - Handles non-linearity through kernels - Robust to outliers	- Computationally intensive for large datasets - Hyperparameter selection required - Sensitive to noisy or overlapping data
Random Forests	- Handles non-linearity and interactions - Robust to noisy data and outliers - Feature importance	- Prone to overfitting with complex models - Limited performance on sparse data - Harder to interpret with deep forests
K-Nearest Neighbors (KNN)	- Simple and intuitive - Captures complex relationships - Robust to noise and outliers	- Sensitive to choice of neighbors (K) - Computationally expensive prediction - Poor performance with high dimensions
Artificial Neural Networks (ANN)	- Captures complex non-linear relationships - Handles various input types - Suitable for large data	- Requires careful hyperparameter tuning - Prone to overfitting with limited data - Lack of transparency
Ridge Regression	- Handles multicollinearity through regularization - Stable and less prone to overfitting	- Requires tuning of regularization parameter - May not perform well with non-linear relationships - No feature selection

**Table 7:** The strengths and weaknesses of each deep learning algorithm used for surface roughness prediction of additively manufactured components.

Technique	Strengths	Weaknesses
Convolutional Neural Networks	- Effective for image data - Captures local patterns and features - Robust to data variations	- Requires large labelled datasets - Limited applicability to non-visual input data
Deep Neural Networks	- Versatile for various data types - Captures complex relationships - Handles structured/unstructured data	- Prone to overfitting - Requires extensive hyperparameter tuning
Generative Adversarial Networks	- Generates synthetic data - Data augmentation potential - Captures intricate patterns	- Challenging and unstable training - Mode collapse potential
Autoencoders	- Captures latent representations - Dimensionality reduction - Data reconstruction	- Struggles with complex non-linear relationships - Interpretability challenges
Deep Reinforcement Learning	- Sequential decision making - Adaptive learning - Optimal strategy discovery	- Computationally intensive training - Reward function design challenges - Training instability and variance

## Data Availability

Not applicable.
